# Influence of Data Sampling Frequency on Household Consumption Load Profile Features: A Case Study in Spain

**DOI:** 10.3390/s20216034

**Published:** 2020-10-23

**Authors:** J. C. Hernandez, F. Sanchez-Sutil, A. Cano-Ortega, C. R. Baier

**Affiliations:** 1 Center for Advanced Studies in Earth Sciences, Energy and Environment, University of Jaén, 23071 Jaén, Spain; jcasa@ujaen.es; 2 Department of Electrical Engineering, University of Jaen, 23071 Jaen, Spain; fssutil@ujaen.es; 3 Department of Electrical Engineering, University of Talca, Camino los Niches Km. 1, Curicó 3344158, Chile; cbaier@ieee.org

**Keywords:** smart meter, temporal data granularity, electric load profile, time slices, time series, advanced metering infrastructure

## Abstract

Smart meter (SM) deployment in the residential context provides a vast amount of data of high granularity at the individual household level. In this context, the choice of temporal resolution for describing household load profile features has a crucial impact on the results of any action or assessment. This study presents a methodology that makes two new contributions. Firstly, it proposes periodograms along with autocorrelation and partial autocorrelation analyses and an empirical distribution-based statistical analysis, which are able to describe household consumption profile features with greater accuracy. Secondly, it proposes a framework for data collection in households at a high sampling frequency. This methodology is able to analyze the influence of data granularity on the description of household consumption profile features. Its effectiveness was confirmed in a case study of four households in Spain. The results indicate that high-resolution data should be used to consider the full range of consumption load fluctuations. Nonetheless, the accuracy of these features was found to largely depend on the load profile analyzed. Indeed, in some households, accurate descriptions were obtained with coarse-grained data. In any case, an intermediate data-resolution of 5 s showed feature characterization closer to those of 0.5 s.

## 1. Introduction

Large temporal datasets for household electricity consumption, provided by smart meters (SMs), offer significant potential for energy time-series scientists. These datasets permit increased resolution and analysis at the level of individual households. Recent studies by Zhou and co-workers [1,2] focus on the challenges and opportunities that SMs provide for smarter energy management where SM data are an essential component.

Traditionally, consumption load metering in the residential context has been conducted at a low time resolution. Thus, consumption profiles are generally gathered for different dwelling types, based on a sampling frequency that provides a data granularity from 1 to 30 min [3]. However, current SCADA systems can sample consumption data at a higher frequency (typically 1 Hz) though standard practice is to store averaged values of 1 min or higher [4].

Reference [5] highlights the importance of taking into account both a wide time slice and frequency spectrum for an accurate description of the load profile features of household consumption.

More specifically, the choice of a temporal data granularity (data sampling frequency) for specifying consumption load profile features has a crucial impact on the results of any action or assessment, as discussed in the literature [6,7,8,9,10,11,12,13,14,15,16,17,18,19,20,21,22,23,24,25,26,27,28,29,30,31,32,33,34,35,36,37,38,39,40,41,42,43,44,45,46,47,48], see Table 1. This table summarizes for each potential action or assessment the time resolution (data granularity) and time horizon (time slice) envisaged for the works related to load profiles in households. The impact of temporal data granularity is important because the consumption profile is known to fluctuate at a high temporal resolution (i.e., interval of 0.01–5 Hz [16,49,50]). Therefore, when a longer time resolution is envisaged, the profile dynamics become increasingly biased. This means that the profile should be sampled at a more fine-grained level, which will more accurately describe its behavior. Nonetheless, there is a trade-off between the computational burden and accuracy of any action or assessment, which is determined by this discrete time resolution [51]. Moreover, the effective measurement of the electrical variables –root-mean-square (RMS) value– requires at most a 5-Hz sampling frequency [52]; a higher frequency would provide just punctual values.

The dynamic nature and frequently high variation typical of household consumption load profiles in the residential context has been analyzed for different temporal data granularities, as shown in Table 1. As disclosed, most of the time resolutions were longer than 1 min. Few references focused on granularities lower than 15 s. High data granularity naturally implies larger amounts of data to be locally stored (either hard disk or memory card) or uploaded to the cloud. This has led many researchers and industry practitioners to develop and survey a vast number of analytical tools that could help to segment and cluster SM big data so that they can be analyzed in real time [11,32]. On the other hand, uploading these data to the cloud (data traffic with the cloud) is another important limitation [11,44,53]. High granularity requires a large bandwidth, which is not always available in individual households. Since data cannot be transmitted at such high resolutions, data compression algorithms are required. The compression ratio can be 10:1 or even 1000:1 can be achieved. For example, Kelly [8] performed measurements every 6.25 × 10^−5^ s(16 kHz) and used the free lossless audio codec (FLAC) compression algorithm that reduced the daily data of 28.8 GB to 4.8 GB with a 6:1 compression ratio. Also, reference [54] developed a new method of data compression via stacked convolutional sparse auto-encoder. These algorithms are usually time consuming, and thus, the gathered data are not available in real time.

The scientific community currently has very limited access to consumption load data in the residential context. The information available for private purposes is either free or must be purchased. Free options are available at different web sites that provide records for homes. Pecan Street [55] is a web site that provides data from 1115 houses with PV and/or EV. Mack [56] developed a web site on SMs for homes, with the aim of saving electricity. Wilcox [53] built a hadoop-scaled SM analytics platform that allows the use of large datasets at a 20 TB scale. Furthermore, there are other web sites that provide household consumption load data with a granularity from 1 s to 1 min [57,58,59,60,61,62,63,64].

Some studies evaluated the feature bias due to the use of coarse-grained data when assessing the consumption load profiles in households. Murray [38] compared time resolution data of 1 min and 15 min and demonstrated the damping effect when working with data with high temporal resolutions in 21 houses in the UK. Naspolini [39] showed that the use of a 15-min data granularity was not well adjusted because of load fluctuations with a period lower than 15 min (5 min) in the operation of electric water heaters. Bucher [40] studied a 1 s and 15-min data granularity in domestic PV-household load profiles. Shi [41] analyzed the accuracy of predictions based on different data granularity. Hoevenaars [64] showed that using a 1-h time step hid the load variability within the hour for models of renewable power systems. Regarding optimization purposes, Van der Meer [42] concluded that a 5-min time resolution provided a good balance between accuracy and the burden of data size, whereas [45] showed that using hourly data led to large biases compared to 1-min data. However, coarser data could be sufficient for household aggregation [48]. A shorter fluctuation at a granularity of only 4 s was investigated in [65].

In this context, it was found that the availability of household consumption load data at a fine granularity (high sampling frequency) obtained from SM measurements allows users to do the following actions or assessments (see Table 1):

- To dynamically adjust electricity tariffs as fine temporal resolutions make it possible to quickly adapt to changes in consumption and thus reduce the electricity bill.
- To improve energy efficiency, comfort, and safety in households with an intelligent automation system.
- To recognize activities (i.e., analysis of energy consumption through observation) that are potentially more meaningful to users.
- To optimally size renewable generation system and storage systems.
- To forecast household load profiles with different time horizons, from a short-term load forecast (hourly and daily) to long-term forecast-based planning studies.
- To perform studies of angle stability, transient analysis, and frequency control in electrical energy systems.
- To characterize household consumption load profile features.
- To perform load forecasting by applying probabilistic techniques.

To date, the major obstacle to accurately describing household consumption load profile features is the fact that this type of profile has not been sampled at temporal low-resolution (high frequency) that gathers its intrinsic dynamics. Thus, most of the examples mentioned in Table 1 used an hourly or even a 30-min or 15-min time resolution, but they do not include enough information to accurately provide actions or assessments. Furthermore, even though the current trend is to use more temporally granular data sets in household applications, the influence of temporal granularity has not as yet been analyzed using a comprehensive and high-resolution data set. Quite a few papers [38,39,40,41,42,45,48,65] evaluated the bias due to the use of coarse-grained data, but never compared resolutions lower than 1 to 5 min. Still another shortcoming is the fact that most studies cover short time slices. These involve a reduced timespan chosen to characterize key aspects of temporal variability, for example, covering weekdays and weekends, different times of day, and different seasons. The time horizon (time slice) envisaged in Table 1 was typically restricted to minutes, a few hours or a few days. However, the resulting description is not accurate since it does not take seasonality into account. Lastly, SM data only reflect a few electrical variables, which means that very little information regarding electrical behavior can be derived (usually energy or power).

To fill this gap, the new methodology presented in this paper makes two major contributions. It first proposes periodograms along with autocorrelation and partial autocorrelation analyses and an empirical distribution-based statistical analysis, which are able to describe household consumption profile features with greater accuracy. This type of analysis reveals key issues about the granularity impact on the load fluctuation, such as the accurate description of its constituent signals. In contrast, the temporal analysis usually found in literature only offers information regarding the granularity impact on the change in the magnitude of the peak and trough load. Secondly, it proposes a framework for data collection in households at a high sampling frequency (>4 Hz) that provides data to be used in the proposed methodology.

A case study of four households in Spain, using thirteen data granularities, from a half-second to 30 min (0.5, 1, 2, 5, 10, 15, 30 s, and 1, 2, 5, 10, 15, and 30 min), provided valuable insights into the influence of data granularity on the description of consumption load profile features. The data set selected, during almost two years, had different consumption features, namely with varying characteristics in terms of the relation between the peak and base load and load fluctuations, which made it possible to take the heterogeneity of real-world load profiles into account. We acknowledge that conducting our analysis with a data sample from four households is a limitation of this study. However, this data sample was adequate to achieve our primary objective of demonstrating the usefulness of the methodology proposed, which was to highlight the information loss regarding the profile features when using coarse-grained data.

The remainder of the paper is organized as follows: Section 2 describes the methodology implemented in this study. Section 3 discusses the results that reflect the influence of data granularity (sampling frequency) on consumption load profile features. Finally, Section 4 presents the conclusions that can be derived from this research.

## 2. Methodology

This section first presents a set of variables for a full description of features for the stochastic dataset derived from household consumption load profiles. The variables are defined from estimated time series models. This is followed by an explanation of the concepts of granularity and time slices. An outline is then provided of the framework for consumption data collection in households at high sampling frequency and its post-processing.

### 2.1. Time-Series Theory

For stationary stochastic data, the theory of time series models provides estimated models, which include the description of the probability mass function (PMF), power spectral density function, and autocorrelation function [66,67].

The features of a stationary stochastic dataset are fully described by the joint probability density function of the observations [66,67]. The joint probability density function of the observations are fully depicted by a stationary stochastic dataset. If the density could be calculated on the basis of observations, then this density would provide all of the information pertaining to the signal. Nevertheless, this is usually not feasible without a great deal of additional knowledge about how such observations were obtained. Features that can always be estimated include the power spectral density and the autocorrelation function. In addition, knowledge of the spectrum (power spectral density function) or autocorrelation (autocorrelation function) along with the first two statistical moments makes it possible to accurately describe the joint probability density function of normally distributed observations [66,67]. Even when assumptions regarding the normal distribution and strict stationarity are not confirmed, previous estimators still provide a sound basis for further research [67]. Nonetheless, in the case of other distributions, higher-order moments provide more information.

#### 2.1.1. Stationary

A time series *x* (and thus the underlying stochastic process) is considered stationary if the process is in a certain state of statistical equilibrium. Accordingly, the properties of a stochastic processes are assumed to be invariant during the translation trough time. This signifies that the joint probability distribution associated with *m* observations $(x_{1},x_{2},\ldots ,x_{m}),$ for any set of time measurements $\left(t_{1},t_{2},\ldots ,t_{m}\right)$, is the same as that for *m* observations $\left(x_{1+k},x_{2+k},\ldots ,x_{m+k}\right)$, at times $(t_{1+k},t_{2+k},\ldots ,t_{m+k}).$ Therefore, the joint distribution must not change when all of the observation times are shifted backward or forward by any integer amount *k*.

Household consumption load profiles are usually not stationary; there is usually daily, weekly, and monthly seasonality and an upward trend as the number of appliances in the household rises. However, as stationary datasets are easier to analyze, there are numerous techniques that can be applied on time series to make it stationary, i.e., transformations, deseasonalisation, and differencing [68].

There are many methods to check whether a time series is stationary or non-stationary: (i) look at plots; (ii) summary statistics; and (iii) statistical tests. The most rigorous approach to detecting stationarity in time series data is using statistical tests developed to detect specific types of stationarity, such as simple parametric models that generate the stochastic process. Among them, it is important to mention the following: (i) Augmented Dickey-Fuller (ADF) test [69]; (ii) Kwiatkowski, Phillips, Schmidt, and Shin (KPSS) test [70,71]; (iii) Variance ratio test [72]; (iv) Leybourne-McCabe (LMC) test [73]; and (v) Phillips-Perron (PP) test [74].

The Dickey-Fuller test was the first statistical test developed to check the null hypothesis that a unit root is present in an autoregressive model of a given time series, and that the process is thus not stationary. An extension of this test (the ADF test) was developed to accommodate more complex models and data. The KPSS test assumes the null hypothesis of stationarity in relation to the linear or average trend, as opposed to the ADF test. While the alternative is the presence of unitary root. The unitary hypothesis represents the stationary nature of the process. The variance ratio test is included in the semi-parametric tests, unlike the two previous ones, which are parametric. The null hypothesis implies non-stationary, and the alternative hypothesis indicates that the process is stationary. El LCM test allows for additional autoregressive lags similar to the ADF test. Although both tests have the same asymptotic distribution, the statistics from the LMC test converge at a higher rate. The PP test states the null hypothesis as the non-stationarity in time series data and the rejection of the unit-root null in favor of the alternative model.

#### 2.1.2. Metrics for Statistics and Probability Analysis

In probability theory, the moments of a stochastic dataset (random variable) ***x*** consist of the expected values of certain functions ***x***. Such variables are a set of descriptive measurements that correspond to the probability distribution of ***x*** and determine whether all the moments of ***x*** are known.

Let ***x*** be a discrete univariate dataset with a finite number of outcomes $\left(x_{1},x_{2},\ldots ,x_{m}\right)$ occurring with probabilities $p_{x}^{}(x_{i}^{})$, i.e., with PMF $p_{x}^{}(x_{i}^{})\left(=P(x=x_{i})]\right)$, its moment of order one, two, and *r* can be specified as follows [75,76]:

(1) $$\begin{array}{l} m_{x}^{1}=E\left[x\right];\;m_{x}^{2}=E\left[x^{2}\right];\;m_{x}^{r}=E\left[x^{r}\right]\\ m_{x}^{r}=\sum_{i=1}^{\infty }x_{i}^{r}\cdot p_{x}^{}(x_{i}^{}) \end{array}$$

The cumulants of a stochastic dataset are variables that constitute an alternative to the moments described in the previous paragraph [76]. Unlike moments, these cumulants cannot be directly obtained by summatory or integrative processes, such as (1). Cumulants can only be found by identifying the moments and applying relationship formulas [75,76]. Accordingly, the first cumulant is the expected value; the second cumulant is the variance; the third cumulants measures asymmetry; and the fourth cumulant measures the tailedness of the probability distribution.

Equation (1) describes how to find the expected value, variance, skewness and kurtosis for discrete random variables according to probability theory. However, some of these variables such as the expected value and variance might strike as very similar to the sample mean and sample variance, respectively, in descriptive statistics. The sample mean and sample variance are random variables because their values depend on what the particular random sample happens to be. In other words, if we know the frequency distribution, or how many times a data value is repeated in the dataset, the following formula can be used to determine the statistics sample mean, $\bar{x},$ and sample variance, $s^{2}$:

(2) $$\begin{array}{l} \bar{x}=\frac{\sum f\cdot x_{i}}{\sum f}\\ s^{2}=\frac{\sum f\cdot {\left(x_{i}-\bar{x}\right)}^{2}}{\sum f} \end{array}$$

Notice that *m* = ∑f is the dataset sample size.

The distinction between variables in statistics and probability analysis is that the statistics vary with each sample dataset, whereas probability variables are fixed when you know the dataset’s probability distribution. The law of large numbers states that as the sample size grows to infinity, statistics provide a more accurate picture of moments of distribution.

#### 2.1.3. Autocorrelation and Spectrum

The covariance between two observations $x_{n}$ and $x_{n+k}$ of a stationary stochastic dataset is formulated as follows:

(3) $$r(k)=\mathrm{cov}(x_{n},x_{n+k})=E\left[\left(x_{n}-\mu_{x}\right)\left(x_{n+k}-\mu_{x}\right)\right]$$

where $\mu_{x}$ is the mean of the dataset.

The quantity r(k) is specified for each integral value of *k*, and the combination of all of these quantities is known as the autocovariance function of $x_{n}.$ It quantifies the covariance between pairs at a distance or lag *k*, for all values of *k*. This signifies that it is a function of lag *k*.

The autocovariance function expresses all knowledge pertaining to Gaussian stochastic data. In combination with the first two statistical moments, it fully characterizes the joint probability distribution function of the data. Only when the distribution significantly departs from normal, it is interesting to study higher-order moments or other features.

In the same way as the covariance between two variables, it is also possible to normalize the autocovariance function r(k) and thus obtain the autocorrelation function ρ(k):

(4) $$\rho (k)=\frac{r(k)}{r(0)}=\frac{r(k)}{\sigma_{x}^{2}}$$

where $\sigma_{x}^{2}$ is the variance of the dataset.

The autocorrelation function reveals how rapidly a signal can change over a period of time. At lag 0, the autocorrelation value is 1. In the case of most physical processes, there is an autocorrelation function that progressively diminishes for greater lags. Accordingly, the relation at a short temporal distance is greater than the relation for longer distances. A long lag value in the autocovariance function is indicative of the slow variation of the data. In contrast, a short lag value signifies that the data at short distances are correlated. Nonetheless, a high value in the autocorrelation function signifies a repetition pattern, and thus reveals a constituent signal in the analyzed dataset. Therefore, the resulting variable that includes the set of high values of the autocorrelation function can provide a means for describing the features of a stationary stochastic dataset.

The partial autocorrelation function, α(k) represents the autocorrelation between $x_{n}$ and $x_{n+k}$ is an indication of $x_{n}$ on $x_{n+k}$ through $x_{n+k-1}$ removed. In the same way, it denotes the autocorrelation between $x_{n}$ and $x_{n+k}$, which is not explained by lags 1 through *k-1*, inclusively [77]:

(5) $$\begin{array}{l} \alpha (1)=corr\left[x_{n+1},x_{n}\right]\;\mathrm{for}\;k=1\\ \alpha (k)=corr\left[x_{n+k}-P_{n,k}\left(z_{n+k}\right),x_{n}-P_{n,k}\left(x_{n}\right)\right]\;\mathrm{for}\;k\geq 2 \end{array}$$

where $P_{n,k}\left(x\right)$ is the surjective operator of orthogonal projection of *x* onto the linear subspace of the Hilbert space spanned by $x_{n+1},\ldots ,x_{n+k-1}$.

There are algorithms for estimating the partial autocorrelation based on the sample autocorrelations [78].

The Discrete Fourier Transform (DFT) of the autocovariance function constitutes the spectrum or power spectral density function h(ω). The Wiener-Khintchine theorem [79,80] defines conditions in which valid autocovariances have a transform that is always non-negative in all contexts; see [81].

(6) $$\begin{array}{ll} h(\omega )=\frac{1}{2\pi }\sum_{k=-\infty }^{\infty }r(k)e^{-j\omega k}, & \;-\pi \leq \omega \leq \pi \\ r(k)=\int_{-\pi }^{\pi }h(\omega )e^{j\omega k}d\omega , & k\;=\;0,\pm 1,\pm 2,\ldots \end{array}$$

The reason why this is known as the ‘power spectral density function’ is evident in the integral for the value *k* = 0:

(7) $$r(0)=\int_{-\pi }^{\pi }h(\omega )d\omega =r(0)=\sigma_{x}^{2}$$

The variance represents the total power in the signal. It is the power spectral density function that provides the distribution of the total power over the frequency range. When the data are characterized by a strong quasi-periodicity with a specific period, they show a narrow peak in the power spectral density instead of one exact frequency, thus revealing a constituent signal in the analyzed dataset. Therefore, the resulting variable that includes the set of narrow peaks of the power spectral density function can provide a means for describing the features of a stationary stochastic dataset.

The fast Fourier transform (FFT) is an algorithm used for computing the DFT more efficiently and faster, described in Equation (6) as an infinite sum. The FFT algorithm systematizes the redundant calculations in a very efficient way, taking advantage of the algebraic properties of the Fourier matrix. The FFT applied to a signal allows thus obtaining its power spectrum, namely, the periodogram of the signal. When a high number of observations (*N*) is involved, the FFT results assure a high accuracy.

When (4) is divided by the variance of the signal, this results in the normalized autocorrelation function ρ(k) and the normalized power spectral density φ(ω):

(8) $$\begin{array}{ll} \varphi (\omega )=\frac{1}{2\pi }\sum_{k=-\infty }^{\infty }\rho (k)e^{-j\omega k}, & \;-\pi \leq \omega \leq \pi \\ \rho (k)=\int_{-\pi }^{\pi }\varphi (\omega )e^{j\omega k}d\omega , & k\;=\;0,\pm 1,\pm 2,\ldots \end{array}$$

### 2.2. Temporal Granularity and Time Slices

Granularity is the temporal resolution of a recorded measurement set, which is used to obtain the variability of the data set, pertaining to a given measurement interval. The time slice is the temporal framework for a given study. The quality of the results depends on the choice of the appropriate granularity and time slice for establishing the household consumption load profile features.

**Figure 1:**
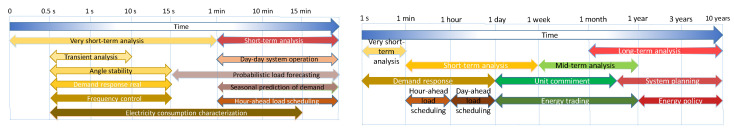
Temporal framework for analyzing the electrical energy system.

The temporal framework for analyzing the electrical energy system differs, depending on the action or assessment performed [19], as shown in Figure 1. Very short-term analysis involves a temporal framework from 0.5 s to 1 min, and mainly includes transient analysis, demand response in real time, angle stability, and frequency control. Short-term analysis is associated with the system operation from various seconds to thirty minutes. It includes day-to-day system operation, hour-ahead scheduling, studies of probabilistic load forecasting, and seasonal prediction of demand. A mid-term analysis involves a temporal framework from various days to one year. This includes maintenance of system assets, unit commitment, energy trading, and energy sales. Finally, long-term analyses, which range from 3 years to more than a decade [19], cover new capacity addition [82], system planning, and energy policies covering future demand growth.

### 2.3. Planned Framework for Consumption Data Collection in Households

**Figure 2:**
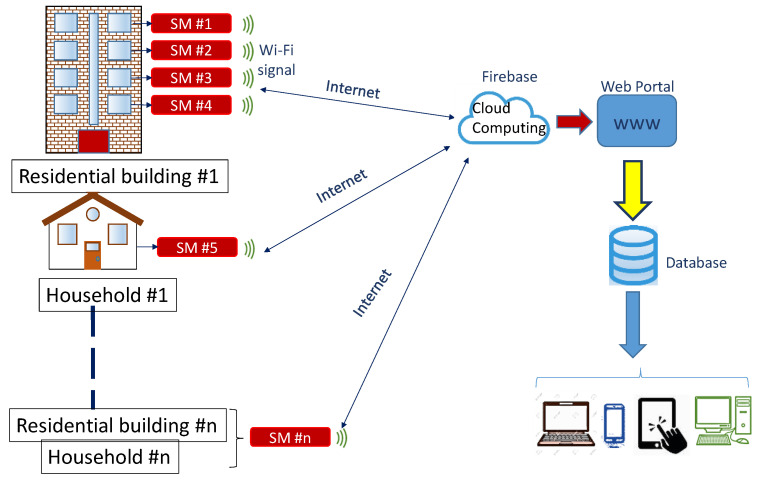
Remote real-time data collection schematic.

Figure 2 shows the planned framework for the remote real-time collection of consumption data at high sampling frequency (>4 Hz) in households with SMs and the uploading of this information to the cloud. This development is one of the most important issues targeted by the SEREDIS project (‘Nuevos servicios de red para microredes renovables inteligentes. Contribución a la generación distribuida residencial’: Grant No. ENE 2017-83860-R [44,49,83,84]). In this framework, the SM designed in [44] is installed in the general protection box, and the data gathered are dumped directly into two data storage solutions: (i) the cloud and (ii) a local storage in SD card.

#### 2.3.1. SM

**Figure 3:**
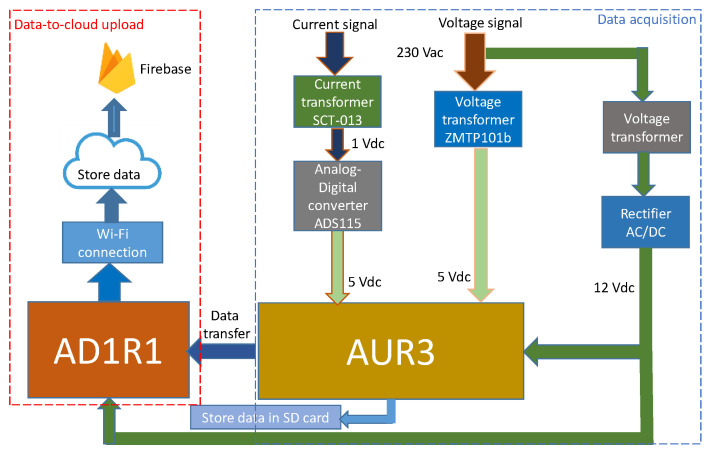
Block diagram for the data acquisition and uploading.

As shown in Figure 3, the SM is composed of a data acquisition block and a data-to-cloud upload block (see [44] for a more in-depth explanation). This SM was calibrated and tested to ensure its reliability and accuracy [44]. This SM has two Arduino boards: (i) the Arduino Uno Rev3 (AUR3 [85]); (ii) the Wemos Arduino D1R1 (AD1R1 [86]). The AUR3 board was used for the measurement process whereas the AD1R1 board uploaded data to the cloud. This reduced the time needed to process data and upload them to the cloud.

The SM simultaneously performed two processes. In the first process, the AUR3 microcontroller software determined the fundamental and derived electrical variables, sent data via serial port to AD1R1, and then stored information in the local data logger. The AD1R1 software read from the serial port and uploaded the data to the cloud via Wi-Fi in a parallel process.

**Figure 4:**
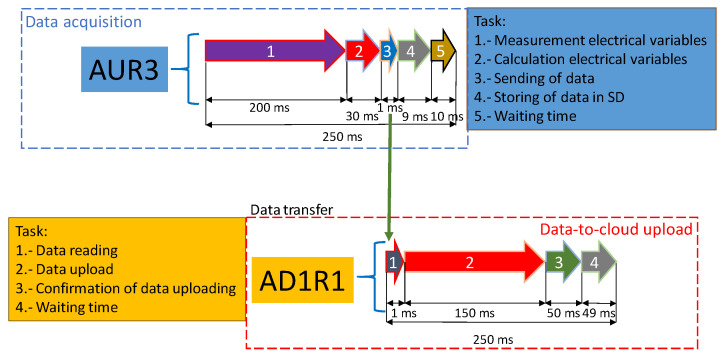
Process timeline for the SM.

The timeline of the processes is shown in Figure 4. In the first process, the required time for measuring electrical variables is a 10-cycle time interval for a 50-Hz power system (Class-A performance [52]). In order to be accurately measured, the frequency of sampling must be at least twice the frequency of the signal. Therefore, 200 samples were used for the 10-cycle interval with a sampling frequency of 1 kHz. Derived variables are then calculated, which takes about 30 ms. The transmission of the information to the serial part only takes 1 ms, and data storage in SD memory, 9 ms. This leaves 10 ms for the waiting time In the second process, the SM reads the data received in 1 ms and uploads the data in 150 ms. About 50 ms are required to confirm the data upload, which leaves 49 ms for the waiting time.

##### Data Acquisition

Figure 3 shows the hardware for the data acquisition process. Analogical voltage and current sensors measured the electrical variables, which were then processed in the Arduino AUR3 [85] microprocessor. Once the fundamental variables were obtained, the derived variables were computed. More specifically, the current sensor STC-013 [87] (of the non-invasive type) together with the voltage sensor ZMPT101b were used for this purpose [88]. A digital/analogue converter ADS1115 was planned to increase the 1V DC output of the current sensor to the 5 V analogue input of AUR3 [89].

##### Data-To-Cloud Upload

The cloud provides a cost-effective method of supporting big data analytics. Therefore, the cloud data storage solution is suitable in scenarios where a real-time response from a given stream of SM data is required. This real-time data availability aids in personalizing applications that benefit both household owners and the scientific community when analyzing consumer profiles.

When data with finer granularities are gathered, the amount of information involved is high and requires data compression algorithms. These algorithms are usually time consuming; thus, a delay between the measurement and the data availability in the cloud appears. To enable a real-time response, this research does not apply compression algorithms, and the time of data-to-cloud upload is set to 0.25 s (see Figure 4), the same as the data acquisition time.

According to Figure 3, the base of the wireless communication module of the SM was the AD1R1 board [88] that acted as the interface between the microcontroller and cloud data storage (i.e., Firebase). The board used the ESP8266 platform as the operation core, which permitted Wired Equivalent Privacy (WEP) or Wi-Fi Protected Access (WPA)/WPA2 () authentication for secure Wi-Fi communication. In addition, it operated with 802.11 b/g/n wireless systems, which were compatible with the majority of the routers and modems on the market. This framework used the platform Firebase [90] to store huge amounts of data from households monitored with IoT technology and cloud computing. Alternatively, wireless communication systems such as 4G and 5G networks can be used. This implies a more expensive data service contract for data-to-cloud uploading.

##### Local Data Storage

A SM is equipped with a SD card mounted on a data logger shield, which is used as a backup to avoid data lost because of data-to-cloud upload problems. The memory size required per household in a year is about 2.2 GB.

### 2.4. Data Post-Processing

In an asynchronous way, data collected can be used for different actions or assessments. In our study, the assessment aims to show the influence of data granularity on the description of consumption load profile features. This required post-processing the data that involved the extraction of data from the planned storage solution and its adaptation for different granularities.

Cloud data were stored in *json* format and could be downloaded at any time. Also, SD card data were stored in *CSV* format and could be downloaded anytime. Therefore, firstly the data of each house in Figure 2 could be downloaded and converted to *CSV* format, if required, resulting in a daily *CSV* file of all electrical measurements. This format could be recognized by applications used for the processing and analysis of data, such as MS Excel and MatLab.

Secondly, the adaptation for different granularities from raw data on a 0.25 s-basis was carried out by the up-sampling method of the RMS value. The data size was reduced in the up-sampling operation. This allowed obtaining data at thirteen resolutions of data granularity, from a half-second to 30 min (0.5, 1, 2, 5, 10, 15, 30 s, and 1, 2, 5, 10, 15, and 30 min).

## 3. Results and Discussion

This section shows the results of the influence of data granularity on the description of household consumption load profile features based on the methodology presented in Section 2. This framework highlighted the information loss regarding the profile features when coarse-grained data were used. We first focus on the temporal results for different time slices from a sub-hourly to a monthly analysis, including daily and weekly analyses. However, the global influence assessment was based on a yearly analysis. This first involved a statistical analysis. After this, periodograms were compared to autocorrelation and partial autocorrelation analyses to highlight significant outcomes regarding profile features.

### 3.1. Case Study

The case study focused on four real-world households in the city of Jaen in southern Spain. As explained, this research is part of the SEREDIS project, which characterized load profiles for household consumption, electric vehicles (EVs), and PV systems in the residential context. Nonetheless, this study is limited to the consumption load in households. The consumption load data came from SM readings as described in Section 2.3, being post-processed as stated in Section 2.4.

The household set selected had different consumption features, namely with varying characteristics in terms of the relation between the peak and base load and load fluctuations, which made it possible to take the heterogeneity of real-world load profiles into account (see Table 2). In addition, important issues determined the household selection, such as the power contracted from the electric mains and the type of supply at home. Single-phase systems were designed at households #1, #2, and #4, whereas household #3 had a three-phase system. The contracted power in Spanish legislation reflects how the household is equipped with different electrical appliances (see Table 3). In this study, the limitation in the number of households was due to a combination of limited financial budget, limited number of households that included EVs and PV systems, and a low number of families who voluntarily cooperated on the research project.

In particular, household #1 was a family flat with three children. Household #2 was a semi-detached house with only two inhabitants. Household #3 was a detached house with two children and their parents. Household #4 was a terraced house of four components, inhabited by two adults and two teenagers.

The city of Jaén has a Mediterranean climate with hot summers but cool winters. Therefore, the houses in our study were all equipped with climate control systems. More specifically, household #1 had a heating system for the whole building with a gas boiler. The flat also had an air-conditioning system for summer. Household #2 had an individual gas boiler for heating, and a two-split air conditioner system (living room and master bedroom). Household #3 was equipped with a central electrical air-thermal system for heating and cooling. Household #4 had an air-conditioning/heat pump system. Each household had different electrical appliances, depending on the age and behavior of the family members, see Table 3.

All of the households had the usual appliances installed in the kitchen. Household #2 did not have a dishwasher. The computer equipment in each household was quite heterogeneous (desktop computer, laptop, smart phone, tablet), and depended on the profession of the occupants, and whether certain members of the household were still students. Moreover, the ages of the occupants also determined the entertainment equipment (TV, stereo system, video games console, etc.). Thus, households #1, #3 and #4 had video game consoles for the children and adolescents that lived there. In contrast, the occupants of household #2 preferred listening to music on a CD/disc or playing movies on DVD/BlueRay. The lighting system in all households was low consumption and high efficiency. Low consumption lamps were planned in households #1, #2 and #3. Households #1, #2 and #4 also had fluorescent lamps. LED lighting was present in households #1, #3 and #4. Only household #4 used halogen lamps.

### 3.2. Reliability of the Planned Framework to Provide Data

**Figure 5:**
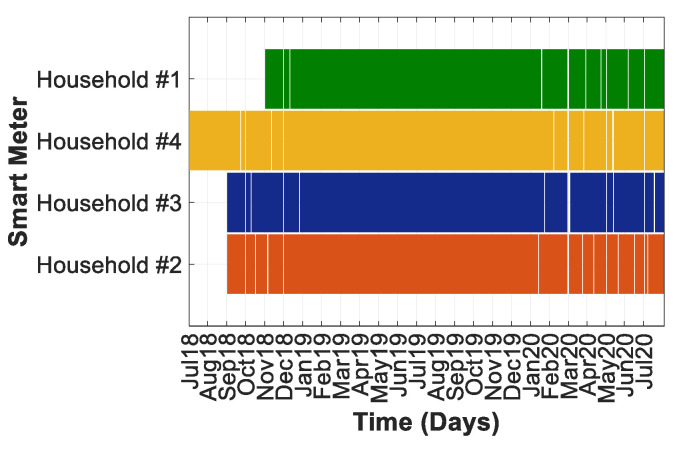
Data availability for every household.

Figure 5 shows the data availability for the data-to-cloud upload in each household within the SEREDIS project up to July 2020. The gaps in the figure represent the days when some information was lost because of data-to-cloud upload problems, particularly when the data loss exceeded 25% in one day. Data collection started in July 2018 (household #3) and is still going on for all of the households. On September 2018, SMs were added to households #1 and #2. Finally, household #4 was equipped with an SM in October 2018.

As data were collected on a 0.25 s basis, 345,600 measurements for six electrical variables (voltage, current, active, reactive and apparent power, power factor) were stored each day. This provided a total data set of 2,073,600 per day, 62,208,000 per month, and 746,496,000 per year.

**Figure 6:**
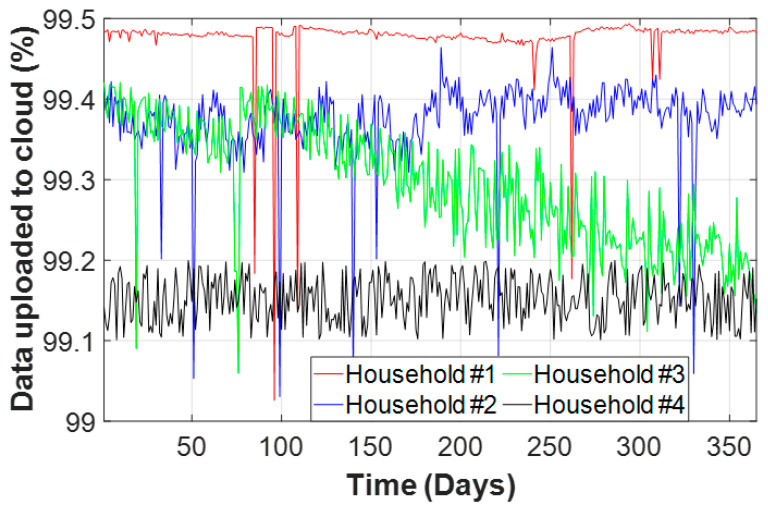
Percentage of successful data-to-cloud upload in 2019.

The reliability of the data-to-cloud upload (Firebase [90]) during 2019 was greater than 99% because of the high quality of the fiber optic Internet connection in all of the households. Figure 6 depicts the percentages of successful data-to-cloud upload.

As explained, the SM also included a local 8-GB memory card that served as a data backup. This permitted an operational autonomy of 1.88 years. This local storage guaranteed 100% data availability; thus, the assessment shown hereafter is based on data of this storage solution.

### 3.3. Sub-Hourly Time Slice Analysis

**Figure 7:**
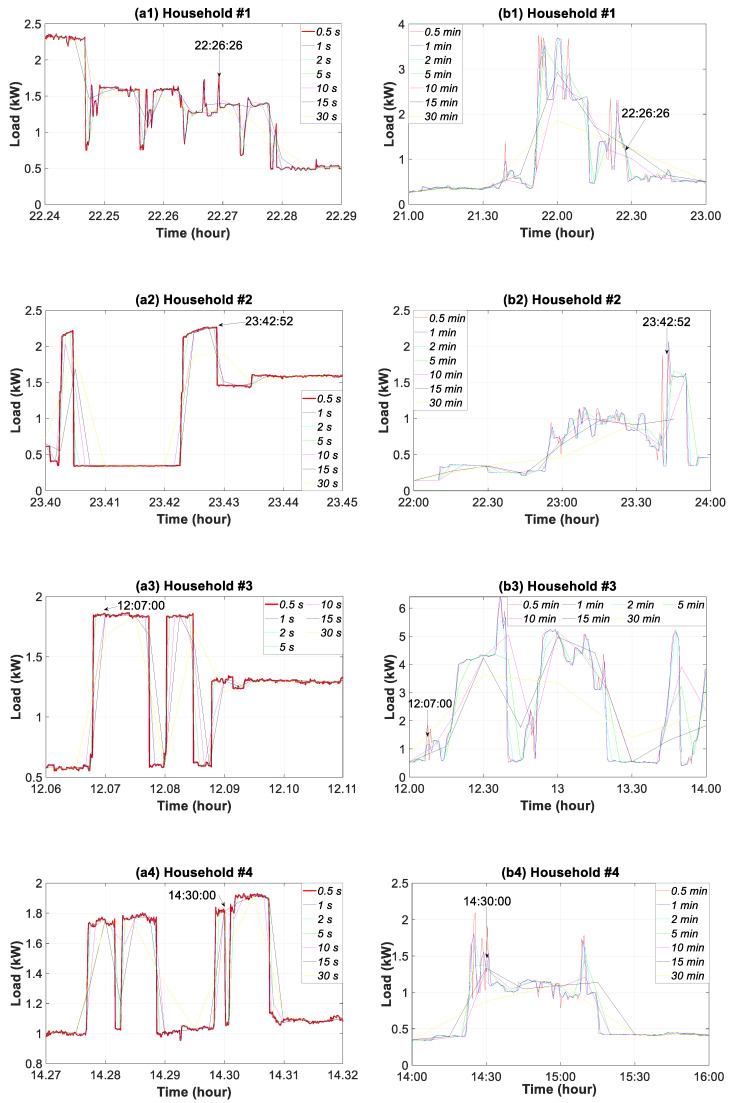
Consumption load profile for household #1 to #4: (**a1**–**a4**) 0.5 to 30 s granularities (5-min timespan); (**b1**–**b4**) 0.5 to 30-min granularities (2-h timespan).

The influence of the data granularity on the temporal change features of the household consumption load profile at sub-hourly level is analyzed in this section. Figure 7 shows the measurements for all of the households and thirteen resolutions of data granularity from 0.5 s to 30 min on a working day in September. Data with finer granularities, from 0.5 s to 30 s, are shown on a 5-min timespan whereas coarser resolutions, from 0.5 min to 30 min, cover a 2-h timespan.

For household #1, Figure 7a1 highlights the smoothing of peaks and troughs in the consumption load because of the use of coarse-grained data. For example, the 1.77 kW load peak at 22:26:26 h was only achieved at a 0.5-s granularity. The granularity of 1 and 2 s gave a load peak of 1.67 kW and 1.61 kW, which meant a decrease of 5.64% and 9.04%, respectively. However, the flattening of this peak was very pronounced for the 30-min granularity where a decrease of 27.12% was observed.

Regarding household #2, the increase in the maximum load peak for the finest granularity in regard to the coarse resolution occurred at 23:42:52 h and was 56.44%. The comparison of the consumption load in household #3 at 12:07:00 h, as an example, shows a reading of 1.83 kW for a 0.5-s granularity, 0.53 kW for a 15 min granularity and 0.81 kW for a 30 min granularity, which meant a decrease of 71.03% and 55.73%, respectively. For household #4, the reading of 1.81 kW for a 0.5-s granularity at 14:30:00 h was reduced to 0.88 kW for the 30-min granularity, which meant a decrease of 51.38%.

Coarse data granularities tend to flatten the peaks and troughs in the consumption load. Since there is a considerable loss of information, the consumption thus evaluated does not conform to reality. The smaller the granularity used, the smoother the load peaks and troughs will be. As a result, the reduction in the measured power is greater, and is thus a less accurate reflection of reality. One problem with reducing the temporal resolution of consumption data sampling is the loss of variability in regard to the intra-temporal steps, which has a particularly high effect on the actions or assessments. The loss of detail observed in Figure 7, does not justify the reduction of the sampling resolution for the consumption load data.

### 3.4. Daily Time Slice Analysis

**Figure 8:**
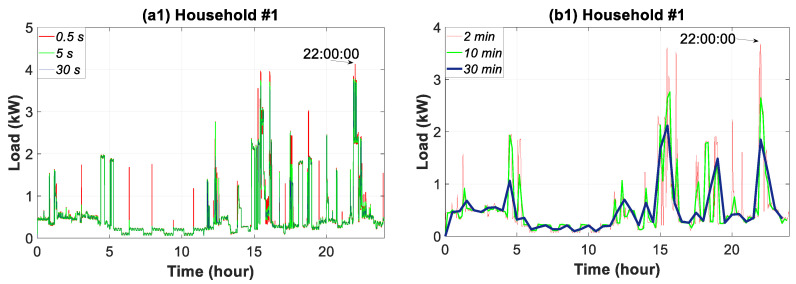

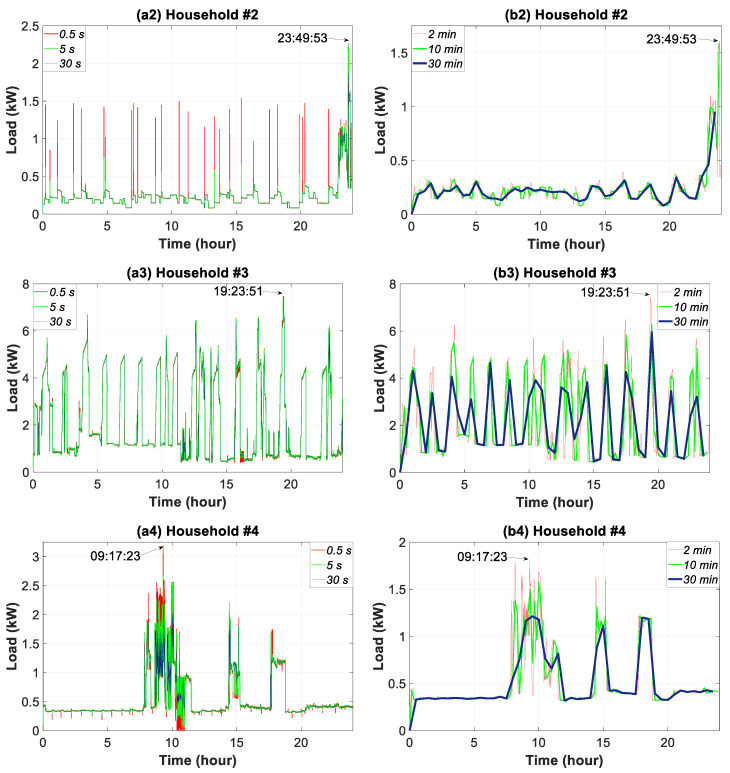
Daily consumption load profile for household #1 to #4: (**a1**–**a4**) 0.5 to 30 s granularities; (**b1**–**b4**) 2 to 30-min granularities.

This section extends the timeframe of Section 3.3 to one day when analyzing the influence of the data granularity. This provides information pertaining to the daily consumption in the households. Figure 8 shows the six samples of data granularity for all of the households on the previously mentioned day of September. This figure highlights the dual nature of the consumption load profile, namely, the continuity of the rough base-load and the intermittent spikes of the peak-load.

For household #1, the maximum daily load peak occurred at 22:00:00 h with a 0.5-s reading of 4.13 kW. However, the readings for the 10 and 30-min granularity dropped to 2.65 and 1.69 kW, respectively, which signified a decrease of 36.07% and 59.09%. As can be observed, the consumption load for household #2 had a sequence of peaks throughout the day for the finest data granularity. These peaks were strongly attenuated for a 5-s data granularity, and much more reduced for the coarse data resolutions. These repeated peaks were caused by the operation of the refrigerator. As an example, the 0.5-s reading at 23:49:53 h was 2.27 kW, after which it dropped to 0.95 kW (a 58.14% decrease) for the 30-min granularity. The central electrical air-thermal system in household #3 originated peaks at a stable timespan. The maximum daily load peak occurred at 19:23:51, with a reading of 7.47 kW for the 0.5-s granularity. In contrast, the 30-min granularity reading dropped to 5.95 kW, a value that was 20.34% lower. In household #4 the maximum daily load peak occurred at 9:17:23 h with a value of 3.14 kW for a 0.5-s granularity, whereas for a 30-min granularity, it decreased by 57.64% (1.33 kW). Repeated peaks were only identified at 0.5 and 5-s granularities during hours when the consumption was lower. These were caused by equipment disconnection. In summary, the greatest loss of information occurred in household #1 (59.07%), followed by #2 (58.14%), #4 (57.64%) and #3 (20.34%).

### 3.5. Weekly Time Slice Analysis

**Figure 9:**
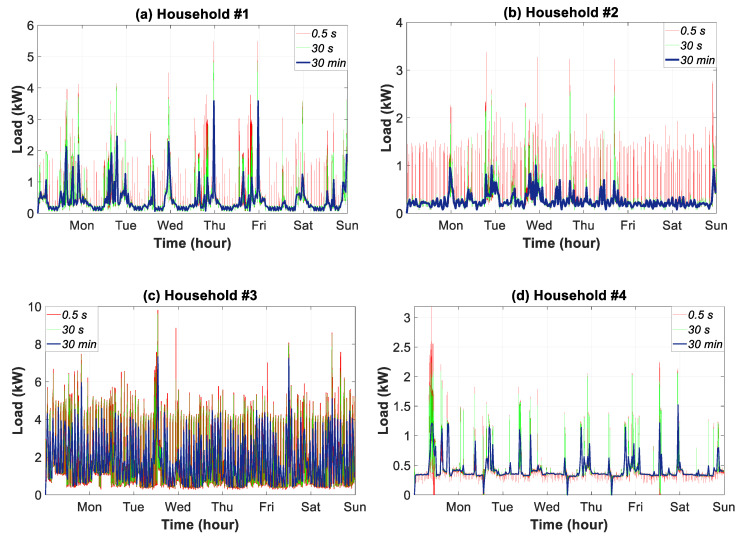
Weekly consumption load profile in households #1 to #4 for three data granularities.

This section discusses the influence of the three samples of data granularity on weekly consumption features, Figure 9. For household #1, the maximum load peaks occurred on Thursday and Friday, with 5.48 kW for the 0.5-s data granularity. These peaks were reduced by 12.77% and 34.67% at the granularities of 30 s and 30 min, respectively. Repeated peaks throughout the day in household #2 were also observed during the weekly analysis. The reduction of peaks by using coarse data granularity, (up to 75%), was the most pronounced in the households analyzed. Intermediate data granularity decreased the weekly load peaks by 37%. In household #3, the highest reduction occurred at the end of Wednesday, which signified a reduction of 30.62% and 72.76% in the temporal resolution of 30 s and 30 min, respectively. The reduction in household #4 was lower, and came to a 62.07% droop for the 30-min data granularity.

### 3.6. Monthly Time Slice Analysis

This section examines the daily smoothing of the highest peak and deepest trough when using coarse-grained data to underline their accuracy. The analysis focused on data from January.

Unlike the remaining sections where the temporal framework and the analysis were very short-term, the results of this section are applicable to medium-term analysis where knowledge of day-to-day operation is required. For this purpose, the ratio between the daily peak or trough load and the daily mean load is used as a metric.

**Figure 10:**
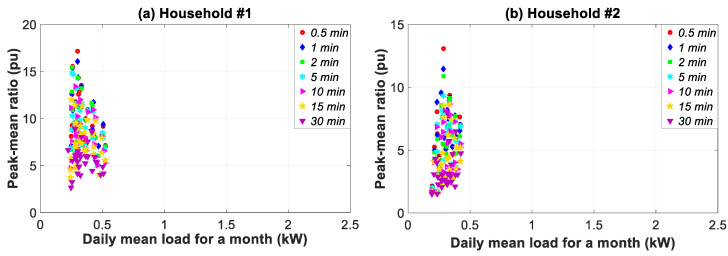

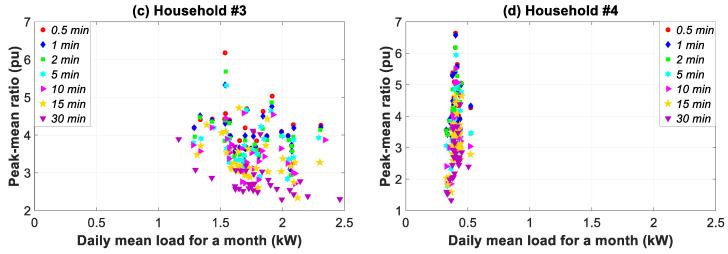
Ratio of daily peak load vs. daily average load in households #1 to #4 for 1.66–30 min data granularity in January.

Figure 10 shows the ratio for the peak load and all of the households for a 0.5–30 min data granularity. The load profiles show a widening spread in the daily mean load for households #3, #1, #2, and #4. This reveals an increase in daily variability as shown in Figure 9.

Table 4 summarizes the highest peak-mean and deepest trough-mean ratios for the thirteen granularities and four households. As can be observed, the monthly maximum peak-mean ratios achieved the highest values for a data granularity of 0.5 s. The ratio decrease for the 30-min data granularity was 50.02%, 39.66%, 35.07% and 36.89% with regard to the 0.5-s granularity, respectively for households #1, #2, #3, and #4. The monthly minimum trough-mean ratio also achieved the highest value for a 0.5-s data granularity. The decrease in percentages with coarser granularity, which was greater than the one for maximum peak-mean ratios, was the following: 64.07%, 75.22%, 27.67% and 59.64%. This lack of accuracy indicates the need to adjust the data granularity at 0.5 s for the medium-term analysis.

### 3.7. Yearly Time Slice Analysis

This section highlights the influence of data granularity on the description of household consumption load profile features by means of different complementary analyses. Firstly, the consumption pattern of each household is justified. Then, based on the use of coarse-grained data, a statistical analysis underlines the change in the annual empirical distribution shape. Finally, periodograms and autocorrelation analyses are used to focus on the loss of information pertaining to profile features, caused by the use of coarse-grained data. This was based on the knowledge of the main constituent signals of the load fluctuations.

#### 3.7.1. Temporal Consumption Pattern

**Figure 11:**
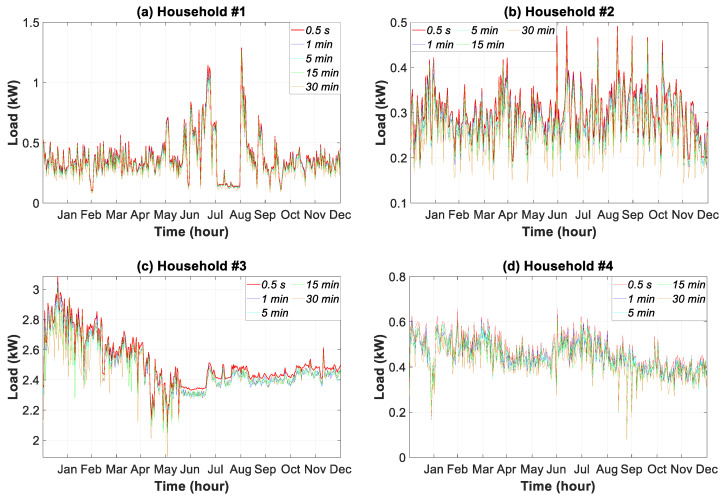
Daily average consumption load profile in households #1 to #4 for five data granularities.

The yearly consumption pattern in a household reflects the energy behavior of the occupants during all seasons, and is strongly influenced by temperature, wind speed, relative humidity, etc. [16,34]. It also takes vacation and holiday periods into account. Accordingly, Figure 11 shows the daily average consumption load during the year for five samples of data granularity in all of the households.

Consumption pattern #1 was stable during spring, autumn, and winter. The heating system in the household was for the whole building, and thus did not influence electricity consumption. However, the air-conditioning system from May to September strongly increased consumption, except for the month of August when the occupants were away on holiday. During the summer (June to September), the children spent more time at home, which increased consumption.

Consumption pattern #2 was stable throughout the year because the occupants were at work all day, and were only at home at night. Nonetheless, there were days between June and October when consumption peaked because of the use of the split air conditioner system.

The central electrical air-thermal system for heating and cooling in household #3 operated the whole year. When temperatures were lower (i.e., January to April), consumption was greater. Regarding household #4, electricity consumption decreased in January and August because the family moved to their second residence.

#### 3.7.2. Statistical Analysis

**Figure 12:**
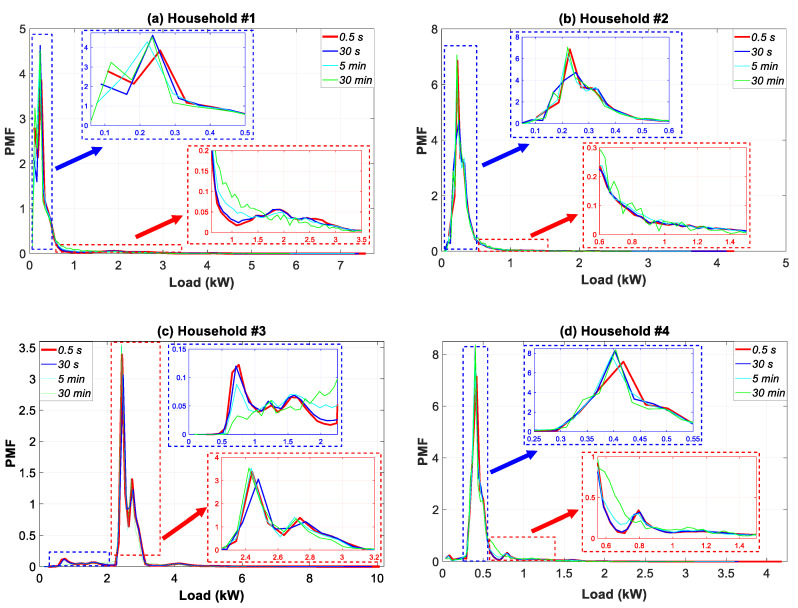
Annual empirical distribution of the consumption load profile in households #1 to #4 for four data granularities.

This section presents a statistical analysis of the datasets monitored in the course of a year (see Section 3.2 for a detailed explanation of the timespan) for the four households in the case study (see Section 3.1), once these datasets were post-processed for different granularities according to Section 2.4. This analysis shows the impact of data granularity on the description of household consumption load profile features. Accordingly, Figure 12 represents the annual empirical distributions (PMFs of the discrete variables) of the consumption load for all of the households and for four data granularities. Specific zooms were included for a better understanding of results.

As can be observed, the PMFs of the household consumption load data are clearly not normal or Gaussian. The most non-Gaussian behavior is evident for household #1, followed by household #3. This result is in consonance with the dual nature of the consumption load profile in Figure 8. In addition, the use of coarser temporal granularity, ranging from 0.5 s to 30 min, substantially affected the PMF shapes. Thus, opposite behaviors were observed. The PMF shape either moved further away from a Gaussian distribution (households #1 and #3) or began to show a more Gaussian behavior (households #2 and #4). In general, the shape was more frequently skewed near those hours with a lower load, which removed many of the extremes. The extreme ends of the PMF were of potential interest as they represented periods of very low or very high consumption.

For profile #1, the PMF moved largely within the intervals of 0.0–0.4 kW and 0.6–3.0 kW. A reduction of occurrences in the 0.0–0.4 kW interval was observed, which moved the higher occurrences towards the 0.6–1.5 kW load interval. In household #3, the lower occurrences in the 0.0–2.5 kW interval were compensated by higher ones in the 2.25–3.20 kW interval. For household #4, a displacement of occurrences from the 0.30–0.55 kW interval to the 0.55–1.50 kW interval was observed.

Table 5 summarizes the statistical results of the annual empirical distributions for all of the data granularities. For purposes of comparison, the values at the 0.5-s data granularity were used as a reference. Using coarser temporal granularity, ranging from 0.5-s to 30-min, led the sample mean of the consumption load to decrease by 17.61%, 17.68%, 17.65%, and 17,74%, respectively for households #1, #2, #3, and #4. In general, the level of variability for all households was significantly reduced as the maximum load values decreased by 15.55%, 18.28%, 13.74%, and 18.49%, respectively. Nonetheless, larger droops were observed for the relevant minimum values, namely, 20.98%, 19.93%, 17.59%, and 16.02%. The reduction in percentage for the variance of all households was in the 27.54–31.29% interval. This again confirmed the drop in the variability of the consumption load for the households.

The sample skewness [91] was positive in households #1, #2, and #3, which underlines that the right tail of the consumption load distribution was longer than the left. On the contrary, household #4 had a negative sample skewness. For coarser data granularities, households #1 and #3 increased their sample skewness value whereas the behavior in households #2 and #4 was exactly the opposite. This was confirmed by the displacement of PMFs in Figure 12.

The sample kurtosis values indicate that all households were leptokurtic [91], which means that the consumption loads were concentrated around the sample mean as household profiles had values greater than 3. Households #1 and #3 had higher sample kurtosis values for coarser granularities, which revealed that the consumption load tended to be closer to the sample mean. This outcome was more pronounced in household #3. However, the behavior in households #2 and #4 was the opposite. Once again, the change in the sample kurtosis values was confirmed by the displacement of PMFs in Figure 12.

#### 3.7.3. Periodogram, Autocorrelation, and Partial Autocorrelation Analyses

This section presents a set of complementary analyses that were performed to explain the influence of data granularity on the description of load profiles. A periodogram analysis, along with an autocorrelation analysis and a partial autocorrelation analysis made it possible to obtain the main periods (or frequencies) of the constituent signals of consumption load fluctuations. The results highlighted the loss of information when using coarse-grained data to describe the load profile features. The analyses were split into two time slices, namely, the 1–100 s interval and the 100 s (1.66 min)–30 min interval. This showed the influence of the aggregation data on these intervals of data granularity. This section concludes by highlighting the daily, weekly, and monthly seasonality of the household consumption load profiles.

Since household consumption load profiles contain more than one source of seasonality, as previously described, our approach at the very beginning removed the trend and seasonal components altogether through differencing and seasonal differencing [68].

To confirm the stationary of the datasets used in periodograms and autocorrelation and partial autocorrelation analyses, Table 6 shows the results the statistical tests described in Section 2.1.1. These tests were applied on the datasets of the annual consumption load profiles for different data granularities. We interpret results using the *p*-value from the test. A *p*-value below a threshold (such as 5%) suggests that we reject the null hypothesis, whereas a *p*-value above the threshold suggests that we fail to reject the null hypothesis.

As can be seen in Table 6, the null hypothesis was rejected in all the tests (*h* = 0 for the tests ADF, variance ratio, LMC, and PP, whereas *h* = 1 for the KPSS test). In addition, the *p*-value analysis for the KPSS and LMC tests yielded reliability levels higher than 99% for most granularities and households. Nonetheless, the reliability levels for the remaining cases were higher than 95%. Therefore, results in Table 6 assured that the differenced time series data were stationary.

**Figure 13:**
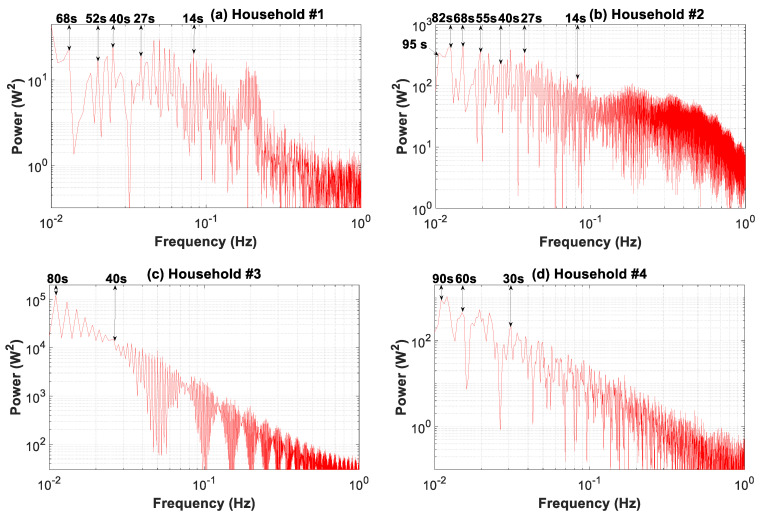
Periodogram of the consumption load profile for households #1 to #4: 1–100 s time slice.

Figure 13 depicts the periodogram of the four consumption load profiles for the 0.5-s data granularity and a period of load fluctuations from 1 to 100 s. The different curves in the graphs were generated with 4000 observations (*N*) drawn on logarithmic scales for better visualization [67]. The property of the autocovariance function assured that its accuracy was proportionally improved with 1/*N*. The accuracy of the periodogram was thus 2.5 × 10^−4^. The finest data granularity of 0.5 s, as described in Section 2, limited the frequency analysis to 1 Hz (10^0^). The power spectral density for load fluctuations showed striking behavior differences for each household. The most stable power spectrum was that of household #1, followed by households #2, #4, and #3. In general, the power spectrum for the four load profiles showed two different patterns. For households #1 and #2, the power level for the 15–80 s interval remained stable. This meant that load fluctuations included several constituent signals of equivalent significance. Thus, main constituent signals at periods of 14, 27, 40, 55, and 68 s can be clearly observed. In contrast, although households #3 and #4 showed various peaks in the periodogram, which corresponded to different constituent signals, their relevance strongly increased with the period rise. This was due to the greater relevance of the induced cycling by the climate control system that masked other minor fluctuating cycles. Nonetheless, some main constituent signals can be observed, such as those at periods of 80 s for household #3, and others at periods of 30, 60, and 90 s for household #4.

These graphs show that when coarse-grained data were used, from 1 to 100 s, there was a loss of information regarding load profile features. This loss was the greatest for household #1, and in descending order of relevance for households #2, #4, and #3. Furthermore, the main constituent signals that reflect this loss of information for coarse data are evident in these graphs. This analysis confirms the results of the information loss in the daily time-slice analysis (Figure 8), where household #1 also showed the worst behavior, followed by households #2, #4, and #3.

**Figure 14:**
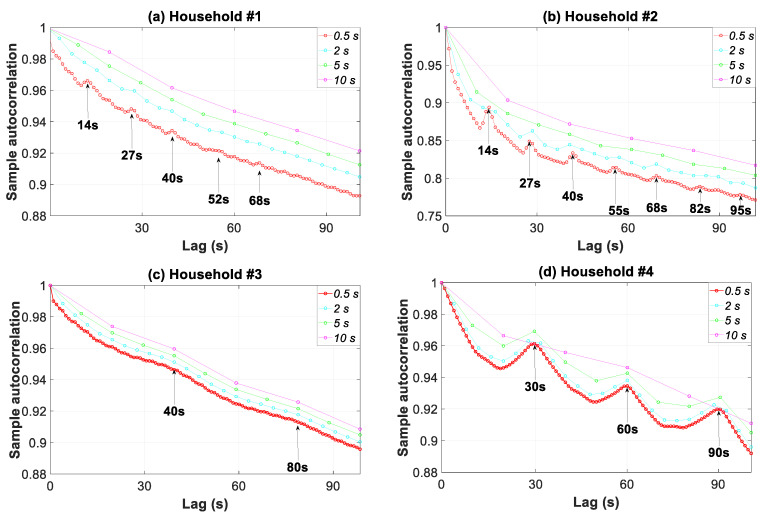
Autocorrelation function of the consumption load profile in households #1 to #4 for four data granularities.

The autocorrelation function, which studies the cross-correlation of a signal with itself, also underlined the constituent signals of load fluctuations. Accordingly, Figure 14 shows the autocorrelation function of the load profiles for four finer levels of data granularity. The curves in the graphs for granularities of 0.5, 2, 5, and 10 s were generated with 200, 50, 20, and 10 observations, respectively. Therefore, the related accuracies were 0.005, 0.02, 0.05, and 0.1, respectively.

A high value in the autocorrelation function signifies a repetition pattern, and thus reveals a constituent signal in the load fluctuation. The first two households had an autocorrelation function that was much lower than 30. For example, in household #1, both the peak autocorrelation values at lags of 14, 27, 40, 52, and 68 s and very strong dips at 11, 25, 38, 50, and 66 s were quite remarkable. The former lags were in consonance with the periods found for the constituent signals in Figure 13a (the periodogram). This outcome is also striking for the other households in Figure 14 when compared with the results in Figure 13.

Figure 14 also underlines the loss of information for the load profile features when using coarser temporal granularity, ranging from 0.5 to 10 s. As the granularity increased, the autocorrelation value at the specific lags moved closer to unity. This unit value meant that data were fully autocorrelated and no different information (different constituent signals) was involved. The higher the granularity was, the greater the loss of information. As an example for household #1 and a lag of 14 s, the autocorrelation value increased by 0.9%, 1,72%, and 2.21% for a data granularity of 2, 5, and 10 s, respectively. As can be observed, the shifting of the curves for each granularity was the highest in household #1, followed by households #2, #4, and #3. This was a consequence of a much ampler and more stable power spectral density level for the 2–10 s period in the constituent signals for household #1 (Figure 13).

**Figure 15:**
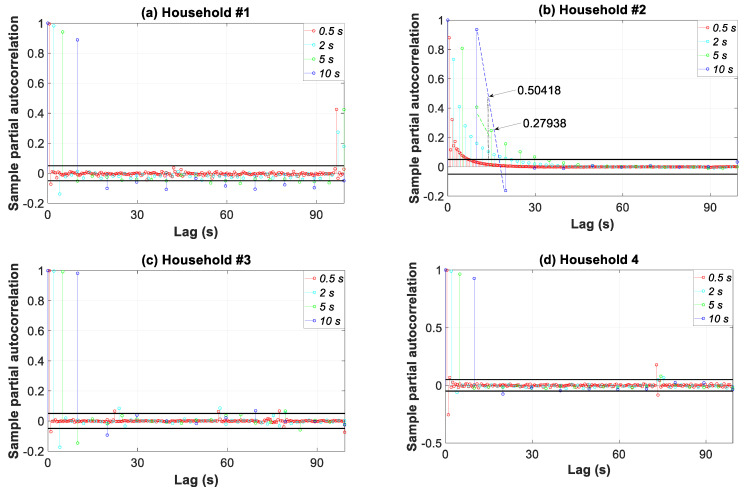
Partial autocorrelation function of the consumption load profile in households #1 to #4 for four data granularities.

The partial autocorrelation function can also be used to underline the loss of information when coarse-grained data were used. Thus, Figure 15 shows the partial autocorrelation function of the load profiles for four data granularities. As an example, the constituent signal of a 14 s period in household #2 was analyzed. As the granularity increased, the partial autocorrelation value at this specific lag moved closer to unity. This unit value meant that data were fully autocorrelated and no different information (different constituent signals) was involved. More specifically, the value of 0.5 s moved from 0.0166 to 0.1031, 0.2794, and 0.5042 for 2, 5, and 10 s, respectively.

**Figure 16:**
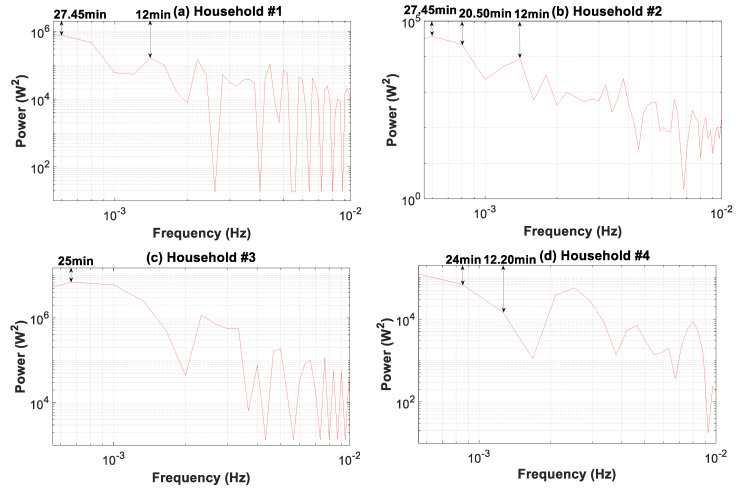
Periodogram of the consumption load profile for households #1 to #4: 1.66–30 min time slice.

Figure 16 broadens the periodogram in Figure 13 covering the period of load fluctuations from 1.66 min to 30 min. The comparison of the power spectral density for the load profiles shows an increasing spread between the base and peak load for profiles #1, #2, #4, and #3. This outcome is evident in Figure 8 and Figure 9, where a drastic reduction of the peaks with higher granularities can be observed. Furthermore, in this interval, the power spectrum level is two orders of magnitude higher compared to that of the 1–100 s interval (Figure 13). Consequently, the contribution to the overall power of the 1–100 s interval was less important and was at least 1% of that of the 1.66–30 min interval.

The power spectrum clearly shows a single constituent signal for household #3, at a period of 25 min, whereas for the remaining households, several main constituent signals are evident. Therefore, in households #1 and #2, there were signals at the 27.45 and 12 min periods, and in household #4, at the 24 and 12.20 min periods. Figure 16 shows that when coarse-grained data from 1.66 to 30 min were used, load profile features were increasingly inaccurate. This lack of accuracy was the greatest for household #1, and in descending order of relevance, for households #2, #4, and #3. Furthermore, the main constituent signals that represent the loss of information for coarse data are shown in Figure 16.

**Figure 17:**
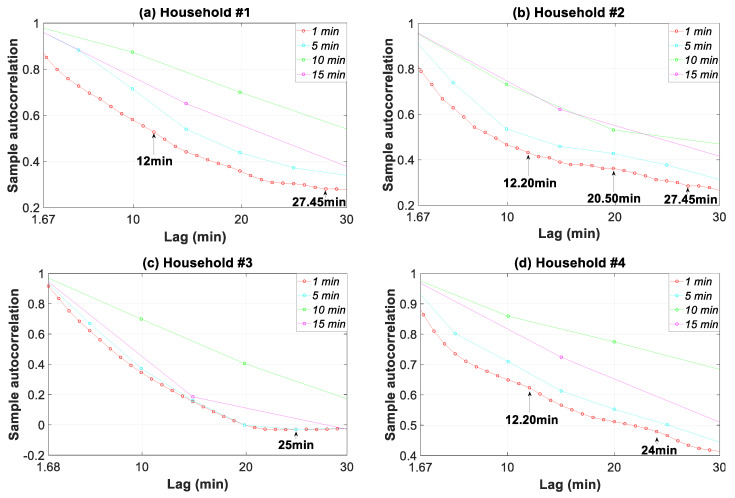
Autocorrelation function of the consumption load profile in households #1 to #4 for four data granularities.

The autocorrelation analysis in Figure 17 pertaining to the 1.66–30 min time slice confirms the constituent signals found in Figure 16 for the different households. In addition, the shifting of the curves (inaccurate profile characterization) for each granularity was in consonance with the power spectral density levels for this 1.66–30 min interval (Figure 16). Thus, the greatest shifting and thus the most inaccurate profile characterization was found in household #1, followed by households #2, #4, and #3.

This section concludes by highlighting the seasonality of household consumption load profiles. However, it is important to note that this seasonality, namely daily, weekly, and monthly, is out of the temporal resolution scope of data granularity analyzed in this study (up to 30 min). Accordingly, raw datasets without applying differencing processes were used.

**Figure 18:**
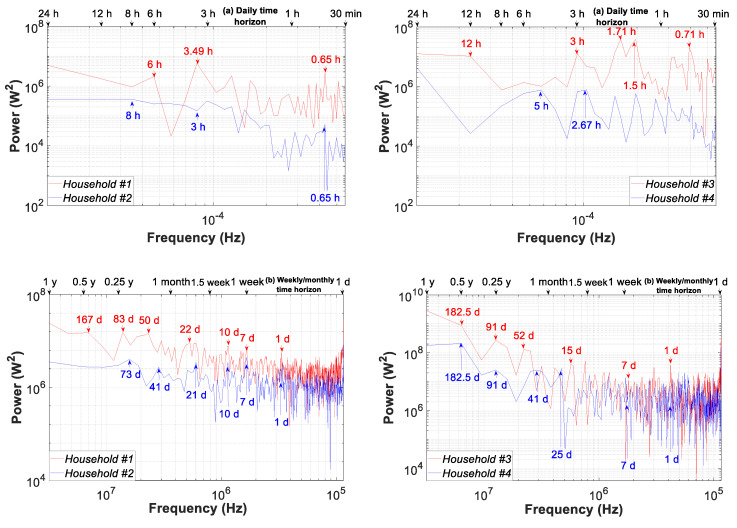
Periodogram of the consumption load profile for households #1 to #4: (**a**) daily time horizon (30 min–1 day time slice); (**b**) weekly/monthly time horizon (1–365 day time slice).

Figure 18 shows the periodograms of the four consumption load profiles for the time horizon daily (a) and weekly/monthly (b). Regarding the daily seasonality in Figure 18a, except for household #2, the power spectrum from the interval of 0.65 h to 24 h had almost the same order of magnitude. For the different households, their main constituent signals can be clearly identified as follows: (i) #1 (0.65, 3.49 and 6 h); (ii) #2 (0.65, 3 and 8 h); (iii) #3 (0.71, 1.50, 1.71, 3, and 12 h); and (iv) #4 (0.92, 1.41, 2.67, and 4.9 h).

Within the time horizon of one year, Figure 18b, the power spectrum shows that cyclic household power levels differ substantially from one month to the next. In addition, the cyclic power in the interval lower than seven days shows striking behavior differences for each household, decreasing in households #1 and #2 and keeping a more stable value in households #3 and #4. For the different households, their main constituent signals during the weekly and monthly time horizons are as follows: (i) #1 (1, 7, 10, 22, 50, 83, and 167 days); (ii) #2 (1, 7, 10, 21, 41, and 73 days); (iii) #3 (1, 7, 15, 52, 91, and 182.5 days); and (iv) #4 (1, 7, 25, 41, 91, 182.5 days).

### 3.8. Comparative Study of Granularity Impact in the Literature

The results obtained in this study were compared with other studies in the literature that address granularity impact. It is important to note that this research not only provides temporal results for different time slices from a sub-hourly to a monthly analysis, but it also offers periodograms along with autocorrelation and partial autocorrelation analyses and empirical distribution-based statistical analysis. The temporal analysis offers information regarding the granularity impact on the change in the magnitude of the peak and trough load. In contrast, the second type of analysis reveals additional information such as the constituent signals in the load fluctuation. Since studies in the literature have focused only on temporal analysis, this comparison was limited to the impact on the magnitude change of the peak load because of the lack of data for autocorrelation analyses and empirical distribution-based statistical analysis.

In this study, the values obtained in the temporal analysis (Figure 7, Figure 8, Figure 9, Figure 10 and Figure 11) and the empirical distribution-based statistical analysis (Figure 12) illustrated that the load peak decreased when the coarse-grained data was compared with the finest data granularity as follows: (i) sub-hourly analysis (between 27.12–56.44%); (ii) daily analysis (between 20.34–59.09%); and (iii) monthly analysis (between 35.07–50.02%).

Wright [27] compared granularities of 30 and 1 min and revealed a peak load reduction between 16–47% for different households. Murray [38] showed peak load changes in the range of 8.9–16% for granularities of 8 s using a meter. Naspolini [39] registered a drop between 18.56–28.36% when 15 min granularity was compared to 5 min granularity. Bucher [40] studied granularities between 5 s and 1 h, which resulted in a reduction between 2–38% as compared to a 1-s based granularity. Shi [41] carried out analysis with granularities of 1, 5, 10, 15, 30, and 60 min and obtained reductions in the peak load of up to 20% (5 min granularity) and up to 80% (60 min granularity) as compared with the 1 min granularity. Widen [48] found drops between 19.19–26.29% for the 60 min granularity with respect to the 10 min. Hoevenaars [64] disclosed a reduction in the peak load when compared to the 1-s based granularity for different granularities as follows: (i) 10 s (between 1.28–7.45% for different sources); (ii) 1 m (between 1.78–15.04%); (iii) 10 min (between 2.46–22.62%); (iv) 60 min (between 8.01–20.65%).

## 4. Conclusions

Increasing interest in the analysis of household electricity consumption profiles, thanks to the rapid deployment of SMs in the residential context, may significantly change the relevance of such profiles in the near future. To understand profile features and their applicability to any action or assessment, it is necessary to appreciate the full range of consumption load fluctuations.

For this purpose, this paper has presented and discussed a methodology that makes two contributions to the state of the art. Firstly, this research proposed periodograms along with autocorrelation and partial autocorrelation analyses and empirical distribution-based statistical analysis, which were used to describe household consumption load profile features. This type of analysis reveals key issues of the granularity impact on the load fluctuation, such as the accurate description of its constituent signals. Secondly, a framework was developed to collect household consumption data at high sampling frequency (>4 Hz). This methodology allowed us to analyze the influence of data granularity on the description of household consumption load profile features. The effectiveness of this methodology was illustrated in a case study of four households in Spain, using thirteen resolutions of data granularity (0.5, 1, 2, 5, 10, 15, 30 s, and 1, 2, 5, 10, 15, and 30 min). We acknowledge that conducting our analysis with this reduced data sample is a limitation of this study. However, it was adequate for achieving our primary objective of demonstrating the usefulness of the proposed methodology in which the ultimate goal was to highlight the information loss regarding the profile features when using coarse-grained data.

Bearing in mind the limits of applicability of our findings, the main outcomes of the study are detailed below. The influence of data granularity on the results for different time slices from sub-hourly to monthly analysis, including daily and weekly analyses, was discussed. Results from sub-hourly analyses highlight the smoothing of peaks and troughs in the consumption load, based on coarse-grained data from 0.5 s to 30 min. More specifically, peaks decreased by 27.12%, 56.44%, 55.73%, and 51.38%, respectively for households #1, #2, #3, and #4. The daily analysis showed higher peak reductions such as 59.09%, 58.14%, 20.34%, and 57.64%, respectively for the previously mentioned households. The repeated peaks were only identified in the daily and weekly analysis at granularities from 0.5 s to 5 s. The monthly analysis provided data pertaining to the day-to-day load behavior by using the ratio between the daily peak or trough load and the daily mean load. This ratio decreased for coarse data granularity by 50.02%, 39.66%, 35.07%, and 36.89%, respectively for households #1, #2, #3, and #4.

However, the overall influence that data granularity had on the description of household consumption load profile features was performed on an annual basis by using a set of complementary analyses. A statistical analysis based on coarse-grained data underlined the significant change in the empirical distribution shape. The analysis of statistical moments up to the fourth-order reflected the reduction of the level of variability of the consumption load for households when coarse-grained data were used. Periodograms and autocorrelation analyses also indicated the loss of information regarding the profile features caused by the use of coarse-grained data. These analyses were based on the main constituent signals of the load fluctuations. In conclusion, the analyses for different granularities showed that some important loads (e.g., cooling or heating devices, electric water heaters, etc.) produced fluctuations that became increasingly ill-suited for resolutions of 5 s or higher. This confirms that coarse granularities should not be used to collect consumption data because they do not reflect the reality.

The results of our study indicate that it is not necessary to use the finest data granularity, i.e., the 0.5-s resolution. In fact, even for profiles #1 and #2, which showed the greatest fluctuation, a data-resolution of 5 s produced a sufficiently accurate characterization of profile features since the results generated were very close to those of a data-resolution of 0.5 s. Therefore, the use of the 5-s granularity achieves a balance between the computational burden associated with storage data in the cloud and their post-processing, and the loss of information for the consumption profile features.

The results in this research were in line with other studies in the literature that address granularity impact. Since studies in the literature focused only on temporal analysis, this comparison was limited to the impact on the magnitude change of the peak load because of the lack of data for autocorrelation analyses and empirical distribution-based statistical analysis. This review offered a peak load reduction between 1.28–80% with granularities in the interval of 1 s to 1 h.

Future work in the field should take the current limitation of this study into consideration. Further analysis could have been conducted with other households of different characteristics, or the methodology could have been applied to a large set of buildings. It is our hope that this study will spur future work and discussion in the research community regarding the accurate description of household load profile features based on an appropriate data granularity and will ultimately lead to similar work on datasets from other multi-family residential buildings.

## Figures and Tables

**Table 1:** Literature published related to load profiles in households.

Time Resolutions (Data Granularity -Sampling Frequency)	Actions or Assessments							Time Horizon (Time Slice)		
	Adjust Tariff Electrics	Energy Efficiency & Comfort	Recognize Activities	Optimization Size Renewable Generation	Electrical Load Forecasting	Power Consumption Characterization	Applying Probabilistic Techniques	A Few Minutes	A Few Hours	A Few Days
<0.5 s	--	--	--	--	--	[8]	--	--	--	--
1 s	--	[8,9,12]	[23]	[13,42]	[12]	[7,8,9,11]	--	[7,13]	[9,23]	[13]
>1 s and <10 s	--	[8,38]	--	[13]	--	[8,38,41]	--	[13]	--	[13]
15 s	--	--	--	[13,14]	[14]	--	--	[13]	--	[13]
1 min	--	[9,12]	[6,12,26]	[13,45]	[12,24,25,26,27,29]	[6,9,27]	--	[19,26,27]	[6,9]	--
5 min	[15]	[15,17]	[26]	[7,16]	[17,18,26,27]	[7,27]	[18]	[15,16,17,18,26,27]	[17]	[17]
15 min	[16]	--	[6,22,26]	[16]	[22,25,26,30,34,37,47]	[6,11,32]	--	[16,26]	[6,33,34,47]	[22]
30 min	--	--	[26]	[42,45]	[22,27,30,33,34,35,42]	[35]	[42]	[26,27,35,42]	[33,34]	[22,35,42]
1 h	--	--	[26,28,36]	[45,48]	[25,26,30,34,36]	[28]	--	[26]	[34,36]	[22]
1 day	--	--	[36]	[43]	[36,37,43]	[37]	--	[37]	[36]	[43]
No available information	[14]		--	[4,40,49]	[19,20,21,29]	[7]	[19,20]		[20]	[19,21]

**Table 2:** Key features of four households in Jaén (southern Spain). Case study.

	Household #1	Household #2	Household #3	Household #4
Total annual consumption (kWh/year)	3033	2626	22,058	4139
Total surface (m^2^)	100	125	210	140
Number of family members	5	2	4	4
Is there at least an adult during the morning at home?	No	No	Yes	Yes
Electric heating	No	No	Yes	Yes
Electric air conditioned	Yes	Yes	Yes	Yes
Building type	Flat	Semi-Detached house	Detached house	Terraced house
Contracted power from the electric mains (kW)	3.45	2.3	5.75	4.6
Number of phases	1-phase	1-phase	3-phase	1-phase

**Table 3:** Appliances and lighting installed in every household case study.

Item	Power (W)	Household #1	Household #2	Household #3	Household #4
Oven	1200–2200	√	√	√	√
Electric cooker	900–2000	√	√	√	√
Extractor hood	70–200	√	√	√	√
Microwave oven	900–2500	√	√	√	√
Dishwasher	1500–2200	√	----	√	√
Refrigerator	250–350	√	√	√	√
Washing machine	1500–2200	√	√	√	√
Electric water heater	1500–5500	----	----	√	----
Vacuum cleaner	1100–2000	√	----	√	----
Dryer	1000–2500	√	√	√	√
Clothes dryer	1500–3000	√	----	√	----
Desktop computer	150–300	√	----	√	----
Laptop	100–250	√	√	√	√
Smart phone	15–25	√	√	√	√
Tablet	20–30	√	----	√	---
LED TV	150–550	√	√	√	√
BlueRay-DVD player	50–75	----	√	----	----
Stereo system	100–150	√	√	----	√
Video games console	25–150	√	----	√	√
Low energy bulbs	5–20	√	√	√	----
Florescent lamps	18–58	√	√	----	√
LED lamps	4–12	√	----	√	√
Halogen lamps	25–60	----	----	----	√

**Table 4:** Monthly maximum/minimum ratio of daily peak-mean load and of daily trough-mean load in January.

Data Granularity	Household #1		Household #2		Household #3		Household #4	
	Maximum Peak-Mean	Minimum Trough-Mean	Maximum Peak-Mean	Minimum Trough-Mean	Maximum Peak-Mean	Minimum Trough-Mean	Maximum Peak-Mean	Minimum Trough-Mean
0.5 s	17.7714	7.4346	13.2673	6.1029	6.3413	3.1745	6.9320	3.2970
1 s	17.4875	7.4233	13.2562	5.4252	6.3114	3.1726	6.9318	3.2951
2 s	17.5749	7.2256	13.2559	4.7885	6.2524	3.1693	6.8127	3.2876
5 s	17.4989	7.0880	13.1816	3.6465	6.2348	3.1668	6.7546	3.2712
10 s	17.3746	6.8418	13.1445	2.3266	6.2191	3.1642	6.7268	3.2671
15 s	17.1912	6.5573	13.0965	2.0043	6.2258	3.1601	6.6947	3.2524
30 s	17.1558	6.5692	13.0793	1.6689	6.1782	3.1554	6.6404	3.2491
1 min	16.0662	6.5542	11.4579	1.6763	5.3288	3.0967	6.5740	3.2495
2 min	15.4650	6.0329	10.8880	1.7029	5.6834	2.9352	6.1811	3.0951
5 min	14.8479	5.0117	9.3116	1.6465	5.3142	2.8401	5.9447	1.7821
10 min	13.4116	4.7214	8.6840	1.6023	4.5818	2.7117	5.5095	1.8488
15 min	12.0069	3.7204	8.6675	1.5882	4.7191	2.3409	4.6373	1.5840
30 min	8.8820	2.6709	8.0044	1.5119	4.1170	2.2958	4.3747	1.3305

**Table 5:** Descriptive statistics of the consumption load profile throughout the year.

Household	Data Granularity	Sample Mean (kW)	Maximum Value (W)	Minimum Value (kW)	Sample Variance (kW^2^)	Sample Skewness (kW^3^)	Sample Kurtosis (kW^4^)
#1	0.5 s	0.3771	1.2884	0.1021	0.0326	1.7935	7.8044
	1 s	0.3752	1.2821	0.1016	0.0323	1.7932	7.8035
	2 s	0.3733	1.2756	0.1011	0.0320	1.7938	7.8060
	5 s	0.3658	1.2497	0.0991	0.0307	1.7930	7.8024
	10 s	0.3628	1.2397	0.0982	0.0302	1.7931	7.8048
	15 s	0.3619	1.2369	0.0980	0.0301	1.7959	7.8179
	30 s	0.3582	1.2247	0.0971	0.0295	1.7940	7.8073
	1 min	0.3545	1.2112	0.0960	0.0288	1.7950	7.8130
	2 min	0.3506	1.1994	0.0950	0.0283	1.7988	7.8334
	5 min	0.3430	1.1722	0.0918	0.0270	1.7963	7.8290
	10 min	0.3388	1.1616	0.0926	0.0264	1.8027	7.8813
	15 min	0.3285	1.1191	0.0884	0.0248	1.7985	7.8953
	30 min	0.3106	1.0882	0.0801	0.0224	1.8782	8.5657
#2	0.5 s	0.2998	0.4912	0.1922	0.0030	0.4923	3.6786
	1 s	0.2983	0.4888	0.1912	0.0028	0.4920	3.6774
	2 s	0.2968	0.4863	0.1903	0.0029	0.4934	3.6812
	5 s	0.2908	0.4764	0.1864	0.0028	0.4927	3.6803
	10 s	0.2884	0.4725	0.1849	0.0028	0.4930	3.6810
	15 s	0.2878	0.4715	0.1845	0.0028	0.4906	3.6731
	30 s	0.2848	0.4668	0.1827	0.0027	0.4913	3.6795
	1 min	0.2818	0.4622	0.1809	0.0027	0.4947	3.6930
	2 min	0.2788	0.4573	0.1790	0.0026	0.4876	3.6675
	5 min	0.2728	0.4488	0.1748	0.0025	0.4886	3.6615
	10 min	0.2701	0.4417	0.1732	0.0025	0.4827	3.6079
	15 min	0.2606	0.4229	0.1683	0.0023	0.4702	3.5331
	30 min	0.2469	0.4014	0.1539	0.0021	0.3548	3.3360
#3	0.5 s	2.5180	3.0848	2.0955	0.0276	0.8327	3.4692
	1 s	2.5054	3.0690	2.0844	0.0273	0.8323	3.4684
	2 s	2.4928	3.0545	2.0745	0.0270	0.8332	3.4696
	5 s	2.4425	2.9928	2.0321	0.0260	0.8329	3.4723
	10 s	2.4224	2.9683	2.0174	0.0255	0.8328	3.4685
	15 s	2.4173	2.9608	2.0108	0.0254	0.8317	3.4663
	30 s	2.3922	2.9311	1.9906	0.0249	0.8312	3.4660
	1 min	2.3670	2.9016	1.9720	0.0244	0.8309	3.4660
	2 min	2.3420	2.8627	1.9485	0.0239	0.8324	3.4470
	5 min	2.2913	2.7917	1.9029	0.0229	0.8126	3.4436
	10 min	2.2669	2.8123	1.8726	0.0225	0.8498	3.5525
	15 min	2.1899	2.6639	1.8024	0.0211	0.7854	3.5506
	30 min	2.0736	2.6612	1.7269	0.0200	1.0132	4.1224
#4	0.5 s	0.4726	0.6576	0.0880	0.0059	−0.5149	4.8532
	1 s	0.4702	0.6544	0.0876	0.0058	−0.5147	4.8532
	2 s	0.4678	0.6510	0.0871	0.0057	−0.5154	4.8545
	5 s	0.4584	0.6379	0.0854	0.0055	−0.5152	4.8533
	10 s	0.4546	0.6323	0.0847	0.0054	−0.5175	4.8579
	15 s	0.4537	0.6314	0.0845	0.0054	−0.5147	4.8602
	30 s	0.4489	0.6251	0.0836	0.0053	−0.5129	4.8537
	1 min	0.4442	0.6179	0.0828	0.0052	−0.5167	4.8618
	2 min	0.4396	0.6133	0.0819	0.0051	−0.5198	4.8590
	5 min	0.4302	0.5983	0.0803	0.0049	−0.5133	4.8487
	10 min	0.4248	0.5972	0.0797	0.0048	−0.5042	4.8242
	15 min	0.4111	0.5617	0.0773	0.0044	−0.5558	4.9013
	30 min	0.3888	0.5360	0.0739	0.0041	−0.4903	4.7124

**Table 6:** Statistical tests to check stationary of the consumption load profiles.

Household	Data Granularity	ADF Test		KPSS Test		Variance Ratio Test		LMC Test		PP Test	
		Null Hypothesis (*H*)	*p*-Value	Null Hypothesis (*H*)	*p*-Value	Null Hypothesis (*H*)	*p*-Value	Null Hypothesis (*H*)	*p*-Value	Null Hypothesis (*H*)	*p*-Value
#1	0.5 s	0	0.0374	1	0.0100	0	1.5166 × 10^−9^	0	0.0100	0	0.0374
	1 s	0	0.0363	1	0.0100	0	6.8206 × 10^−8^	0	0.0100	0	0.0363
	2 s	0	0.0472	1	0.0100	0	5.6420 × 10^−7^	0	0.0100	0	0.0472
	5 s	0	0.0455	1	0.0100	0	6.2874 × 10^−11^	0	0.0100	0	0.0455
	10 s	0	0.0436	1	0.0100	0	1.4630 × 10^−7^	0	0.0100	0	0.0436
	15 s	0	0.0433	1	0.0174	0	7.1719 × 10^−6^	0	0.0100	0	0.0433
	30 s	0	0.0499	1	0.0100	0	2.6792 × 10^−40^	0	0.0100	0	0.0399
	1 min	0	0.0399	1	0.0100	0	4.9340 × 10^−10^	0	0.0100	0	0.0399
	2 min	0	0.0379	1	0.0100	0	0.0001	0	0.0100	0	0.0379
	5 min	0	0.0279	1	0.0100	0	0.0458	0	0.0100	0	0.0279
	10 min	0	0.0456	1	0.0478	0	0.0215	0	0.0181	0	0.0456
	15 min	0	0.0158	1	0.0100	0	0.0036	0	0.0100	0	0.0158
	30 min	0	0.0183	1	0.0213	0	0.0184	0	0.0100	0	0.0183
#2	0.5 s	0	0.0010	1	0.0100	0	0.0410	0	0.0100	0	0.0010
	1 s	0	0.0224	1	0.0100	0	0.0328	0	0.0100	0	0.0224
	2 s	0	0.0010	1	0.0100	0	0.0321	0	0.0100	0	0.0010
	5 s	0	0.0152	1	0.0100	0	0.0320	0	0.0100	0	0.0152
	10 s	0	0.0446	1	0.0100	0	0.0306	0	0.0100	0	0.0446
	15 s	0	0.0470	1	0.0100	0	0.0341	0	0.0100	0	0.0470
	30 s	0	0.0387	1	0.0100	0	0.0412	0	0.0100	0	0.0387
	1 min	0	0.0401	1	0.0100	0	0.0152	0	0.0100	0	0.0401
	2 min	0	0.0408	1	0.0100	0	0.0117	0	0.0100	0	0.0408
	5 min	0	0.0434	1	0.0100	0	0.0399	0	0.0100	0	0.0434
	10 min	0	0.0446	1	0.0310	0	0.0324	0	0.0100	0	0.0446
	15 min	0	0.0475	1	0.0386	0	0.0320	0	0.0100	0	0.0475
	30 min	0	0.0470	1	0.0100	0	0.0483	0	0.0100	0	0.0470
#3	0.5 s	0	0.0302	1	0.0100	0	0.0223	0	0.0100	0	0.0302
	1 s	0	0.0299	1	0.0100	0	0.0332	0	0.0100	0	0.0299
	2 s	0	0.0361	1	0.0100	0	0.0135	0	0.0100	0	0.0361
	5 s	0	0.0357	1	0.0100	0	0.0442	0	0.0100	0	0.0357
	10 s	0	0.0331	1	0.0100	0	0.0443	0	0.0100	0	0.0331
	15 s	0	0.0330	1	0.0100	0	0.0447	0	0.0100	0	0.0330
	30 s	0	0.0329	1	0.0100	0	0.0477	0	0.0100	0	0.0329
	1 min	0	0.0314	1	0.0100	0	0.0206	0	0.0100	0	0.0314
	2 min	0	0.0338	1	0.0100	0	0.0379	0	0.0100	0	0.0338
	5 min	0	0.0417	1	0.0100	0	0.0464	0	0.0100	0	0.0417
	10 min	0	0.0265	1	0.0333	0	0.0426	0	0.0100	0	0.0265
	15 min	0	0.0151	1	0.0100	0	0.0150	0	0.0100	0	0.0151
	30 min	0	0.0415	1	0.0100	0	0.0408	0	0.0100	0	0.0415
#4	0.5 s	0	0.0298	1	0.0100	0	1.6278 × 10^−30^	0	0.0100	0	0.0298
	1 s	0	0.0263	1	0.0100	0	7.7177 × 10^−20^	0	0.0100	0	0.0263
	2 s	0	0.0429	1	0.0100	0	5.5778 × 10−219	0	0.0100	0	0.0429
	5 s	0	0.0491	1	0.0100	0	1.9919 × 10^−52^	0	0.0100	0	0.0391
	10 s	0	0.0459	1	0.0100	0	0	0	0.0100	0	0.0459
	15 s	0	0.0444	1	0.0100	0	4.1505 × 10−150	0	0.0100	0	0.0447
	30 s	0	0.0436	1	0.0229	0	4.8221 × 10^−9^	0	0.0100	0	0.0364
	1 min	0	0.0451	1	0.0719	0	4.3992 × 10^−22^	0	0.0100	0	0.0418
	2 min	0	0.0449	1	0.0450	0	2.2699 × 10^−7^	0	0.0100	0	0.0498
	5 min	0	0.0458	1	0.0100	0	6.6692 × 10^−18^	0	0.0100	0	0.0482
	10 min	0	0.0451	1	0.0100	0	0.0155	0	0.0238	0	0.0413
	15 min	0	0.0482	1	0.0100	0	0.0051	0	0.0100	0	0.0421
	30 min	0	0.0389	1	0.0100	0	0.0384	0	0.1000	0	0.0399
